# Effect of intraoperative dexmedetomidine on hepatic ischemia-reperfusion injury in pediatric living-related liver transplantation: A propensity score matching analysis

**DOI:** 10.3389/fsurg.2022.939223

**Published:** 2022-07-27

**Authors:** Liang Zhang, Ling-Li Cui, Wen-He Yang, Fu-Shan Xue, Zhi-Jun Zhu

**Affiliations:** ^1^ Department of Anesthesiology, Beijing Friendship Hospital, Capital Medical University, Beijing, China; ^2^ Division of Liver Transplantation, Department of General Surgery, Beijing Friendship Hospital, Capital Medical University, Beijing, China; ^3^ Clinical Center for Pediatric Liver Transplantation, Capital Medical University, Beijing, China; ^4^ Liver Transplantation Center, National Clinical Research Center for Digestive Diseases, Beijing, China

**Keywords:** dexmedetomidine, ischemia-reperfusion injury, liver transplantation, pediatrics, postreperfusion syndrome

## Abstract

**Background:**

Hepatic ischemia-reperfusion injury (HIRI) is largely unavoidable during liver transplantation (LT). Dexmedetomidine (DEX), an *α*2-adrenergic agonist, exerts a variety of organ-protective effects in pediatric populations. However, evidence remains relatively limited about its hepatoprotective effects in pediatric living-related LT.

**Methods:**

A total of 121 pediatric patients undergoing living-related LT from June 2015 to December 2018 in our hospital were enrolled. They were classified into DEX or non-DEX groups according to whether an infusion of DEX was initiated from incision to the end of surgery. Primary outcomes were postoperative liver graft function and the severity of HIRI. Multivariate logistic regression and propensity score matching (PSM) analyses were performed to identify any association.

**Results:**

A 1:1 matching yielded 35 well-balanced pairs. Before matching, no significant difference was found in baseline characteristics between groups except for warm ischemia time, which was longer in the non-DEX group (44 [38–50] vs. 40 [37–44] min, *p* = 0.017). After matching, the postoperative peak lactic dehydrogenase levels decreased significantly in the DEX group than in the non-DEX group (622 [516–909] vs. 970 [648–1,490] IU/L, *p* = 0.002). Although there was no statistical significance, a tendency toward a decrease in moderate-to-extreme HIRI rate was noted in the DEX group compared to the non-DEX group (68.6% vs. 82.9%, *p* = 0.163). Patients in the DEX group also received a significantly larger dosage of epinephrine as postreperfusion syndrome (PRS) treatment (0.28 [0.17–0.32] vs. 0.17 [0.06–0.30] µg/kg, *p* = 0.010). However, there were no significant differences between groups in PRS and acute kidney injury incidences, mechanical ventilation duration, intensive care unit, and hospital lengths of stay. Multivariate analysis revealed a larger graft-to-recipient weight ratio (odds ratio [OR] 2.657, 95% confidence interval [CI], 1.132–6.239, *p* = 0.025) and intraoperative DEX administration (OR 0.333, 95% CI, 0.130–0.851, *p* = 0.022) to be independent predictors of moderate-to-extreme HIRI.

**Conclusion:**

This study demonstrated that intraoperative DEX could potentially decrease the risk of HIRI but was associated with a significant increase in epinephrine requirement for PRS in pediatric living-related LT. Further studies, including randomized controlled studies, are warranted to provide more robust evidence.

## Introduction

Hepatic ischemia-reperfusion injury (HIRI) is generally unavoidable during liver transplantation (LT) and can trigger increases in liver enzyme levels, early allograft dysfunction (EAD), and even primary nonfunction (PNF) (1, 2). It has been shown that HIRI is significantly associated with an increased risk of postoperative morbidity and mortality following LT (2–4). Despite recent improvements in pharmacological interventions and surgical techniques, HIRI during LT is still a critical issue that needs to be resolved in clinical practice.

Dexmedetomidine (DEX), a selective *α*2-adrenergic agonist with sedative, analgesic, anxiolytic, and sympatholytic properties, is increasingly used in pediatric clinical practice (5). Despite limited clinical data, the intravenous administration of DEX as an anesthetic adjuvant to provide organ-protective effects during the perioperative period of LT has attracted great attention. In 2016, Fayed et al. (6) first described the hepatoprotective effects of DEX in adult living-related LT, i.e., intraoperative DEX improved postoperative liver graft function. Subsequently, researchers from the Tianjin First Center Hospital have demonstrated that intraoperative DEX administration protected against myocardial, kidney, and brain injuries in pediatric living-related LT (7–9). More recently, Zhang and colleagues (10) observed that intraoperative low-dose DEX administration was associated with reduced HIRI in pediatric deceased LT. However, mixed results have been reported in the literature (11–13), and some researchers (11) failed to detect a benefit of perioperative DEX infusion on postoperative cognitive dysfunction in adult living-related LT.

To date, no research assessed the influence of intraoperative DEX use on postoperative liver graft function or the severity of HIRI in pediatric living-related LT recipients. Thus, this retrospective propensity score matching (PSM) study was designed to provide further evidence for the roles of intraoperative DEX in pediatric living-related LT.

## Methods

### Data source and study population

**Figure 1:**
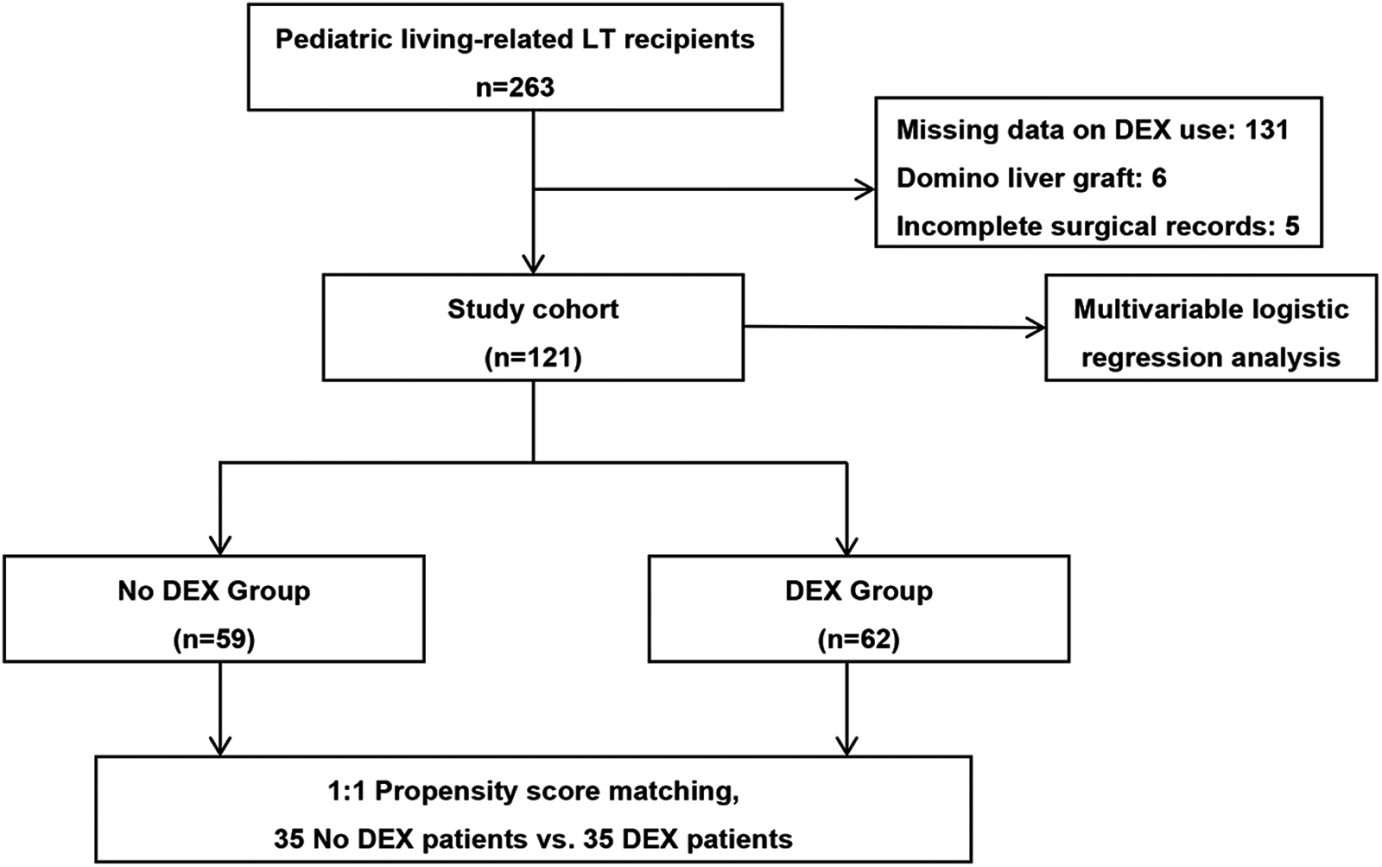
Patient and analysis flowchart. Of the patients, 142 were excluded due to missing data related to DEX use, implanted with a domino liver graft, or incomplete surgical records. DEX, dexmedetomidine; LT, liver transplantation.

This study was a single-center, retrospective cohort study conducted at the Beijing Friendship Hospital, which is one of the three largest pediatric LT centers in mainland China. A database of all pediatric patients who underwent LT from June 2015 to December 2018 was reviewed. Figure 1 shows the selection process of the study subjects. Patients aged less than 16 years who underwent living-related LT were initially screened. The exclusion criteria included patients with missing data related to DEX use, patients implanted with a domino liver graft, and patients without complete surgical records.

### Clinical practice and data collection

DEX was first licensed in mainland China in 2009 but was not approved for perioperative use in our hospital until 2017. Thus, the patients included in this study were classed into one of the following two groups: (1) non-DEX group, i.e., early practice group without intraoperative DEX, and (2) DEX group, i.e., recent practice group with intraoperative DEX. As previously described (14), all patients in the early practice group received a standardized anesthesia protocol. Since June 2017, in addition to the standardized anesthesia protocol, the patients were administrated a continuous infusion of DEX (Aibeining®; SINGCH PHARM., Jiangsu, China) at 0.4 µg/kg/h without a loading dose from incision to the end of surgery. In our hospital, the surgical procedures of pediatric living-related LT have been standardized, i.e., a modified piggyback technique with total clamping of the inferior vena cava was adopted in all cases. Postoperatively, all patients were transferred to the intensive care unit (ICU) for ongoing monitoring and postoperative care and were weaned from mechanical ventilation according to the standard ICU protocols of our hospital.

Preoperative data, intraoperative details, and postoperative outcomes were retrospectively collected from case notes and electronic databases. The collected data mainly included age, sex, height, weight, indications for LT, Child–Pugh score, Pediatric End-stage Liver Disease (PELD) score, graft weight, graft-to-recipient weight ratio (GRWR), cold ischemia time (CIT), warm ischemia time (WIT), postoperative alanine aminotransferase (ALT), aspartate aminotransferase (AST), lactic dehydrogenase (LDH), blood urea nitrogen (BUN), and serum creatinine (sCr) levels within seven days after surgery, occurrences of postreperfusion syndrome (PRS), moderate-to-extreme HIRI, and acute kidney injury (AKI), duration of mechanical ventilation, and ICU and hospital lengths of stay.

### Study endpoints

The primary endpoints were postoperative liver graft function and HIRI severity. The secondary outcomes were postoperative kidney function and the occurrence of PRS and AKI. The severity of HIRI was determined using a modified Rosen’s classification based on the postoperative peak ALT, AST, and LDH levels within 72 h post-LT, i.e., mild (<600 IU/L), moderate (600–1,999 IU/L), severe (2,000–4,999 IU/L), or extreme (>5,000 IU/L) (15). PRS was diagnosed based on Aggarwal’s definition when systolic arterial pressure fell below 70% of the baseline value for at least 1 min within 5 min of reperfusion (16). AKI was defined based on the sCr criteria per the Kidney Disease: Improving Global Outcomes (KDIGO) classifications within seven days post-LT (17).

### Ethical aspects

The study was in accordance with the principles of the Declaration of Helsinki, and the study protocol was approved by the Institutional Review Board of the Beijing Friendship Hospital (Approval number 2020-P2-043-02). Because of the retrospective nature of the study design, the requirement for written informed consent from patients was waived. This study follows the Strengthening the Reporting of Observational Studies in Epidemiology (STROBE) statement for cohort studies (18).

### Statistical analyses

The continuous variables are presented as the mean (± standard deviation) or median (25th–75th percentile), and the intergroup comparisons were carried out using Student *t* tests or Mann–Whitney *U* tests, based on the data distribution. The categorical variables are described as counts (%), and the intergroup comparisons were performed by a Pearson's chi-squared test or Fisher's exact test when more than 20% of cells with an expected count of less than five were observed. Regarding the statistical adjustment for differences in the baseline characteristics, one-to-one PSM models within a caliper set at 0.02 were constructed based on each patient's estimated propensity score (according to age, sex, height, body weight, Child–Pugh score, PELD score, graft type, graft weight, GRWR, type of preservation solution, CIT, and WIT). To identify the independent risk factors of moderate-to-extreme HIRI, potentially significant variables with a *p* value <0.1 in the univariate analysis were further evaluated by multiple regression using a forced entry method. All statistical analyses were performed using SPSS version 22.0 (SPSS Inc., Chicago, IL, USA). A *p* value <0.05 was considered statistically significant.

## Results

### Patient characteristics

In total, 121 pediatric patients who underwent living-related LT met the inclusion criteria. The characteristics of the unadjusted and PSM study subjects are provided in Table 1. In our study, only WIT statistically differed between the groups in the unadjusted comparison (44 [38–50] vs. 40 [37–44] min, *p* = 0.017). Using propensity scores, 35 patients receiving intraoperative DEX were successfully matched to 35 patients without intraoperative DEX (Figure 1). There were no significant differences in the baseline characteristics between the two PSM groups.

**Table 1:** Baseline characteristics of study patients before and after propensity score matching.

Variables	Before matching			After matching		
	No DEX (*n* = 59)	DEX (*n* = 62)	*p-*value	No DEX (*n* = 35)	DEX (*n* = 35)	*p-*value
Age, y	1.3 (0.6–3.8)	1.3 (0.6–3.6)	0.785	1.8 (0.7–4.0)	0.8 (0.6–4.0)	0.267
Female sex, *n* (%)	30 (50.8)	31 (50.0)	0.926	18 (51.4)	18 (51.4)	1.000
Height, cm	75 (69–95)	77 (67–96)	0.957	82 (70–95)	71 (67–98)	0.553
Weight, kg	10.0 (7.8–14.5)	9.6 (7.0–15.0)	0.868	10.5 (7.0–15.0)	9.0 (6.8–13.0)	0.694
Child–Pugh score	8 (6–10)	7 (5–9)	0.061	7 (5–9)	8 (5–10)	0.550
PELD score	10 (−7–20)	6 (−8–18)	0.237	1 (−8–16)	14 (−8–18)	0.315
Type of graft, *n* (%)			0.394			0.320
Segment II	7 (11.9)	3 (4.8)		6 (17.1)	2 (5.7)	
Left lateral lobe	45 (76.3)	51 (82.3)		25 (71.4)	28 (80.0)	
Left lobe	7 (11.9)	8 (12.9)		4 (11.4)	5 (14.3)	
Graft weight, g	255 (220–302)	248 (205–288)	0.185	262 ± 57	266 ± 68	0.779
GRWR, %	2.55 (2.02–3.17)	2.47 (1.66–3.38)	0.495	2.63 ± 0.96	2.73 ± 0.97	0.661
Preservation solution, *n* (%)			0.051			0.607
HTK	45 (76.3)	37 (59.7)		23 (65.7)	25 (71.4)	
Celsior	14 (23.7)	25 (40.3)		12 (34.3)	10 (28.6)	
Cold ischemia time, min	61 (50–90)	56 (41–74)	0.148	60 (42–78)	49 (38–72)	0.157
Warm ischemia time, min	44 (38–50)	40 (37–44)	0.017	40 (37–45)	39 (36–42)	0.359

Data are expressed as mean (standard deviation), median (interquartile range), or number (percent) as appropriate. DEX, dexmedetomidine; GRWR, graft-to-recipient weight ratio; HTK, Histidine-Tryptophan-Ketoglutarate; PELD, Pediatric End-stage Liver Disease.

### Study outcomes before and after the PSM analyses

**Figure 2:**
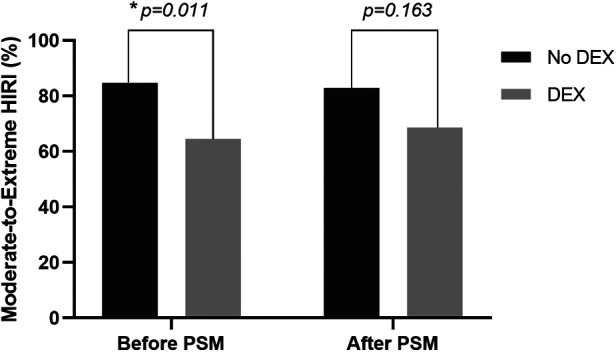
Comparison of the incidence of moderate-to-extreme hepatic ischemia-reperfusion injury (HIRI) between patients with and without intraoperative dexmedetomidine (DEX) before and after the propensity score matching (PSM) analysis. The *p* values were calculated using the Pearson's chi-squared test. *p < 0.05.

In the unadjusted analyses, the incidence of moderate-to-extreme HIRI and postoperative peak serum ALT and LDH levels in the DEX group were much lower than that in the non-DEX group (64.5% vs. 84.7%, *p* = 0.011; 470 [344–623] vs. 579 [393–893] IU/L, *p* = 0.036; 626 [512–899] vs. 968 [639–1,392] IU/L, *p* < 0.001, respectively) (Table 2). After PSM, only the postoperative peak level of serum LDH in the non-DEX group was significantly higher than that in the DEX group (970 [648–1,490] vs. 622 [516–909] IU/L, *p* = 0.002). There was a tendency toward decreased moderate-to-extreme HIRI in the DEX group, but this was not statistically significant (68.6% vs. 82.9%, *p* = 0.163) (Table 2 and Figure 2). Furthermore, the epinephrine dosage used for the treatment of PRS in the DEX group was significantly higher than that in the non-DEX group (0.28 [0.17–0.32] vs. 0.17 [0.06–0.30] µg/kg, *p* = 0.010). However, there was no significant difference between the two PSM groups in the occurrences of PRS and AKI, duration of mechanical ventilation, ICU and hospital lengths of stay, or other outcomes (Table 2).

**Table 2:** Comparison of study outcomes before and after propensity score matching.

Variables	Before matching			After matching		
	No DEX (*n* = 59)	DEX (*n* = 62)	*p*-value	No DEX (*n* = 35)	DEX (*n* = 35)	*p*-value
Primary outcomes						
Peak AST, IU/L	731 (427–1,026)	573 (407–850)	0.158	676 (431–1,026)	602 (429–849)	0.485
Peak ALT, IU/L	579 (393–893)	470 (344–623)	0.036	584 (335–872)	521 (328–733)	0.421
Peak LDH, IU/L	968 (639–1,392)	626 (512–899)	<0.001	970 (648–1,490)	622 (516–909)	0.002
Moderate-to-Extreme HIRI, *n* (%)	50 (84.7)	40 (64.5)	0.011	29 (82.9)	24 (68.6)	0.163
Second outcomes						
PRS, *n* (%)	50 (84.7)	57 (91.9)	0.217	29 (82.9)	34 (97.1)	0.106
Epinephrine dosage for PRS, µg/kg	0.18 (0.10–0.26)	0.25 (0.16–0.32)	0.007	0.17 (0.06–0.30)	0.28 (0.17–0.32)	0.010
Peak BUN, mmol/L	6.8 (5.0–8.0)	5.2 (4.4–6.4)	0.012	6.7 (4.8–8.0)	5.1 (4.5–6.1)	0.066
Peak sCr, µmol/L	39.1 (32.4–48.6)	35.8 (23.7–45.5)	0.157	39.1 (32.3–48.2)	35.1 (22.9–45.4)	0.124
Acute kidney injury, *n* (%)	4 (6.8)	4 (6.5)	1.000	1 (2.9)	1 (2.9)	1.000
Ventilation time^a^, hours	3 (2–5)	2 (1–4)	0.159	3 (2–5)	3 (2–4)	0.730
ICU stay^a^, hours	90 (65–112)	90 (68–112)	0.699	91 (68–116)	89 (70–113)	0.846
Hospital stay^a^^,^^b^, days	31 (23–54)	27 (21–37)	0.027	32 (23–58)	28 (23–37)	0.145

Data are expressed as mean (standard deviation), median (interquartile range), or number (percent) as appropriate. ALT, alanine aminotransferase; AST, aspartate aminotransferase; BUN, blood urea nitrogen; DEX, dexmedetomidine; HIRI, hepatic ischemia-reperfusion injury; ICU, intensive care unit; LDH, lactic dehydrogenase; PRS, postreperfusion syndrome; sCr, serum creatinine.

^a^ Patients with preoperative mechanical ventilation (two patients in each group) were excluded from the final analysis.

^b^ Patients who died during the early postoperative period (one patient in each group) were excluded from the final analysis.

### Predictors of moderate-to-extreme HIRI in pediatric living-related LT

According to the univariable analyses, the potential predictors of moderate-to-extreme HIRI in the overall population were age (odds ratio [OR] 0.867, 95% confidence interval [CI], 0.758–0.992, *p* = 0.037), height (OR 0.982, 95% CI, 0.965–1.000, *p* = 0.047), weight (OR 0.938, 95% CI, 0.883–0.997, *p* = 0.039), graft type (OR 2.625, 95% CI, 0.861–8.002, *p* = 0.090), GRWR (OR 2.060, 95% CI, 1.225–3.463, *p* = 0.006), and intraoperative DEX administration (OR 0.327, 95% CI, 0.136–0.789, *p* = 0.013). According to the multivariate logistic regression analysis, GRWR was a precipitating factor for the presence of moderate-to-extreme HIRI (OR 2.657, 95% CI, 1.132–6.239, *p* = 0.025), while intraoperative DEX administration (OR 0.333, 95% CI, 0.130–0.851, *p* = 0.022) was a protective factors for the presence of moderate-to-extreme HIRI (Table 3).

**Table 3:** Univariate and multivariate logistic regression analysis assessing the risk factors for moderate-to-extreme HIRI in pediatric living-related liver transplantation.

Variables	Univariate		Multivariate	
	OR (95% CI)	*p*-value	OR (95% CI)	*p*-value
Age (per y)	0.867 (0.758–0.992)	0.037	0.729 (0.448–1.186)	0.203
Gender (Male vs. Female)	0.553 (0.240–1.271)	0.163		
Height (per cm)	0.982 (0.965–1.000)	0.047	1.045 (0.954–1.145)	0.348
Weight (per kg)	0.938 (0.883–0.997)	0.039	1.076 (0.818–1.414)	0.602
Child–Pugh score	1.136 (0.946–1.365)	0.172		
PELD score	1.016 (0.987–1.045)	0.282		
Preservation solution (HTK vs. Celsior)	0.821 (0.347–1.941)	0.653		
Type of graft (LL vs. LLL)	2.625 (0.861–8.002)	0.090	3.231 (0.567–18.402)	0.186
Graft weight (per g)	1.004 (0.997–1.010)	0.294		
GRWR (per %)	2.060 (1.225–3.463)	0.006	2.657 (1.132–6.239)	0.025
Cold ischemia time (per min)	0.992 (0.981–1.003)	0.144		
Warm ischemia time (per min)	1.012 (0.964–1.063)	0.626		
DEX administration (Yes vs. No)	0.327 (0.136–0.789)	0.013	0.333 (0.130–0.851)	0.022

CI, confidence interval; DEX, dexmedetomidine; GRWR, graft-to-recipient weight ratio; HIRI, hepatic ischemia-reperfusion injury; HTK, Histidine-Tryptophan-Ketoglutarate; LL, Left lobe; LLL, Left lateral lobe; OR, odds ratio; PELD, Pediatric End-stage Liver Disease.

## Discussion

Our results indicate that intraoperative DEX might provide some protection against HIRI in pediatric living-related LT after adjusting for potential confounding factors. Furthermore, an infusion of DEX at 0.4 µg/kg/h without a loading dose was not associated with delayed postoperative recovery. In contrast to expectations, however, DEX administration did not decrease the risk of PRS but was associated with an increased dosage of epinephrine for PRS treatment. To the best of our knowledge, this study is the first to assess the effect of intraoperative DEX on HIRI in pediatric LT using a matched case-control design.

In the available literature, several criteria (15, 19–22) have been proposed to quantify the extent of HIRI in LT recipients. However, the peak serum AST levels within 24–72 h post-LT remain the most commonly documented indicators. In contrast to clinical trials, serum LDH is generally used as a surrogate marker of HIRI in animal experiments (23, 24). Considering the relatively mild HIRI severity in living-related LT, the modified Rosen's criteria (15) were used to quantify the severity of HIRI in the present study. HIRI, which is an inherent complication of LT, is a complex pathological process that involves the two distinct phases of hepatic ischemic insult and subsequent reperfusion injury (1–3). HIRI associated with LT has frequently led to elevated liver enzymes, EAD, PNF, and graft rejection (1–4). EAD following LT represents the most common form of HIRI with a reported incidence ranging from 20% to 40% and is unequivocally associated with increased post-LT morbidity and mortality (3). To date, the responsible mechanisms, including the release of inflammatory cytokines and chemokines, the generation of oxygen free radicals, the activation of Kupffer cells and neutrophils, the increased expression of adhesion molecules, and infiltration by circulating lymphocytes and/or monocytes, are complex and not well understood (1, 2). Thus, hepatoprotective strategies in the setting of clinical LT and attempts to elucidate the pathophysiology of HIRI are urgently warranted.

Hitherto, several pharmacological strategies have been proposed to protect against HIRI in the setting of LT. More recently, Ito et al. (25) found that the pre-LT long-term administration of rifaximin, which is a broad-spectrum antibiotic and an anti-inflammatory agent against gut-derived hepatic inflammation, exhibited a hepatoprotective effect with a reduced incidence of EAD after adult deceased LT. In a randomized trial of 99 adult LT patients, Bharathan et al. (26) demonstrated that the perioperative administration of prostaglandin E1 significantly decreased the incidence of postoperative AKI and the peak serum levels of sCr and ALT after adult living-related LT. Despite the increasing research focus, the impact of N-acetylcysteine on liver graft function in patients undergoing LT has remained controversial for over two decades (27, 28). In animal models of LT, both ulinastatin and glutathione have been reported to exert a protective effect against HIRI (23, 24, 29). However, there remains a paucity of effective pharmacological strategies to protect against HIRI in the setting of pediatric LT.

DEX is a highly selective alpha-2-adrenergic agonist that is being increasingly used in pediatric practice (5). In the non-general anesthesia scenario, DEX is most commonly administered as the sole sedative agent because it does not cause respiratory depression and can mimic an arousable and physiological sleep state. In the general anesthesia scenario, DEX is often used as an anesthetic adjuvant due to its anesthetic-sparing effects. Over the past decade, numerous clinical studies have documented the multi-organ protective effects of perioperative DEX against organ damage (30). In accordance with the present results, Fayed and colleagues demonstrated that an intraoperative infusion of DEX at 0.8 µg/kg/h could exert hepatoprotective effects against HIRI in adult living-related LT recipients, as shown by an improved liver graft function, better histopathological scores, and decreased ICAM-1 levels (6). A recent retrospective study showed that intraoperative low-dose DEX was associated with reduced HIRI in pediatric deceased LT (10). Another ongoing randomized controlled trial (NCT03770130) is currently investigating the effects of intraoperative DEX compared to placebo on EAD and PNF in adult deceased LT (31).

Despite this emerging interest, the exact mechanisms underlying the hepatoprotective effects of DEX are not fully understood. In general, the hepatoprotective effects of DEX have primarily been attributed to its anti-inflammatory properties. A recent review article further outlines the potential mechanisms by which DEX exerts its hepatoprotective effects, including the downregulation of ICAM-1 expression, the inhibition of iNOS activity, and reductions in the levels of catecholamines, endothelin-1, TNF-α, and IL-10 (32). In animal models, several signaling pathways, including the TLR-4/NF-*κ*B (33), NLRP3 (34), NLRC5 (35), PPARgamma/STAT3 (36), and GSK-3β/MKP-1/Nrf2 signalings (37), have been shown to be responsible for the hepatoprotective actions of DEX.

Notably, previous studies also indicated that a larger GRWR is associated with worse postoperative liver graft function (38, 39). Despite the controversy, it is generally accepted that PRS is the first manifestation of HIRI immediately after graft reperfusion (40). Furthermore, it has been shown that alleviating HIRI by pharmacological agents (41, 42) or machine perfusion (43, 44) can reduce the occurrence of PRS. Counterintuitively, however, the present study failed to demonstrate a preventive effect of DEX on PRS. In contrast, the intraoperative DEX administration was associated with a more significant amount of epinephrine necessary for PRS treatment. These findings are consistent with those reported by Fayed and colleagues (6), who found that the use of DEX increased the dosage of intraoperative vasopressors in adult living-related LT recipients. However, DEX has been demonstrated to reduce vasopressor requirements in the setting of septic shock (45–47). The mechanisms underlying these inconsistencies are unclear, but we propose several possible reasons. The heterogeneity of the enrolled patients (e.g., the severity of shock of the patients) may represent a major reason for these discrepancies. It is well known that DEX can decrease sympathetic activity and circulating catecholamine levels, but its catecholamine-sparing effect exists only when there is a desensitization and downregulation of adrenergic receptors, which are not commonly observed in the setting of living-related LT (47–49). A further explanation for the inconsistencies among these studies might be that the dosage and duration of the DEX administration substantially differed across the studies. It has also been reported that higher doses of DEX are associated with hypertension by directly stimulating *α*1-adrenergic receptors (49).

The present study indicates that, at least in pediatric living-related LT recipients, the use of intraoperative DEX is not significantly associated with improved postoperative outcomes but is associated with decreased HIRI, which may be partially due to the limited sample size in this retrospective and monocentric study; further studies are needed to determine whether intraoperative DEX affects postoperative outcomes, including postoperative complications, length of stay, and in-hospital mortality. Notably, the intraoperative administration of DEX at 0.4 µg/kg/h without a loading dose was found to be well tolerated by pediatric LT recipients, did not result in any serious adverse reactions, and did not prolong the time to awakening and extubation. Nevertheless, DEX should not necessarily be routinely used in pediatric LT recipients without limits. Potential adverse reactions, such as bradycardia (50), hypotension (5), hypertension (5), elevated blood glucose (51), decreased serum potassium (51), drug accumulation (52), and interaction with tacrolimus (53), still need to be monitored particularly closely in clinical practice.

There are several possible limitations in this study that deserves special attention. First, this study is a retrospective matched study. Undoubtedly, unknown confounders that can influence the results likely exist; even consecutive cases were recruited to eliminate selection bias as soon as possible. Second, as the results of this study were obtained from a single center with a limited sample size and all patients received the same anesthesia care, the generalizability of our findings is uncertain. Third, there was no objective assessment of the possible advantages and side effects of DEX use in pediatric patients with living-related LT, such as its impacts on anesthetic requirements, intraoperative hemodynamic stability, serum potassium and glucose levels, and post-LT delirium. Fourth, as severe HIRI is relatively uncommon in living-related LT, the hepatoprotective efficacy of DEX obtained in this study may have been subject to selection bias. Fifth, only a single dose of intraoperative DEX was used in this study. Thus, whether the hepatoprotective effect of DEX in pediatric living-related LT is dose-dependent and whether the prolonged duration of DEX administration into the postoperative period could enhance the protective effects of DEX against HIRI following LT remain important questions that could not be answered in this study. Sixth, this study had a small sample size. Intravenous DEX might potentially provide a protective effect against HIRI but did not significantly change the occurrence of PRS, duration of mechanical ventilation, and ICU and hospital length of stay. Thus, whether the favorable effect of intraoperative DEX against HIRI can be translated to clinical benefits in terms of the postoperative outcomes of pediatric LT patients with living-related LT remains unclear. Finally, some unnoticed changes in surgical and anesthetic practices may have interfered with the primary outcomes over the three-year study period. Further clinical studies, especially randomized controlled trials with a large sample, are still needed to address the above issues. If further studies show a consistent beneficial effect of perioperative DEX on clinical outcomes following pediatric living-related LT, the implications for clinical practice could be immense.

In conclusion, intraoperative DEX use was associated with a lower incidence of moderate-to-extreme HIRI, reduced serum LDH levels, and an increased amount of epinephrine for the treatment of PRS. Furthermore, greater GRWR and intraoperative DEX were independent predictors of moderate-to-extreme HIRI in pediatric living-related LT. Overall, the results and underlying mechanisms should be corroborated by future randomized clinical trials.

## Data Availability

The original contributions presented in the study are included in the article/Supplementary Material, further inquiries can be directed to the corresponding author/s.
